# Innovation Network Reconfiguration Makes Infrastructure Megaprojects More Resilient

**DOI:** 10.1155/2022/1727030

**Published:** 2022-09-15

**Authors:** Ruijiao Sun, Yisheng Liu, Jianghu Zhao

**Affiliations:** ^1^School of Economics and Management, Beijing Jiaotong University, Beijing 100044, China; ^2^Beijing General Municipal Engineering Design & Research Institute Co., Ltd., Beijing 100082, China

## Abstract

Innovation management of infrastructure megaprojects is a challenging task. There are many risks in the process of innovation in engineering technology, such as shortage of funds, policy fluctuations, and difficulties in the transformation of achievements. Meanwhile, innovation organizations involve multiple participants, which makes cooperation complicated. Therefore, resilient innovation is proposed and considered as a tool that can optimize innovation management. The resilience of innovation depends largely on partnerships at the organizational level, which is rarely explored in current studies. This research aims to examine the relationship between organizational resilience and innovation network characteristics. Based on a survey of 164 participants in infrastructure innovation projects, the structural equation model (SEM) is used to explore the factors that influence organizational resilience. The findings show that there is a positive correlation between network characteristics and organizational resilience. Furthermore, the strength of network connections has a direct impact on the preventive and resistance ability of resilience. Network heterogeneity has an impact on the dual ability of resilience. Finally, a case study of the Qinghai-Tibet Railway innovation network shows that based on the above influence paths, we can find a strategy to reconstruct the network to improve resilience.

## 1. Introduction

Innovation is perhaps more important today than ever before [1]. With the rapid and complex changes taking place in the environment of life and business operation, the traditional construction industry needs continuous innovation in materials, machinery, management, and other aspects to move toward high performance, digitalization, and intelligence. This kind of multi-dimensional breakthrough innovation is often found in infrastructure megaprojects. Infrastructure megaprojects (hereafter, we use the term “megaprojects”) are large-scale and complex projects that require substantial innovations during their planning, design, construction, and delivery stages [2]. Megaprojects include different stakeholders who can affect or be affected by project implementation. These stakeholders have to collaborate by sharing knowledge and information to innovate the socio-technical systems, making necessary innovations to solve construction problems and achieve sustainability further [2, 3]. Megaprojects always get more funding for science and technology innovation research because there is more government intervention in these projects. Enterprises have strong desires to participate in complex project innovation, which can not only provide sufficient financial support to innovators but also keep close contact with relevant government departments, to gain more resource accumulation [4]. A strong willingness to participate prompts owners, contractors, designers, universities, and other parties to form partnerships, join the innovation organization, and form an innovation cooperation network [5, 6].

However, innovation organizations of infrastructure megaprojects (IOIM) are facing unprecedented risks, such as the global climate crisis, energy crisis, and political crisis. In particular, since 2019, the outbreak of COVID-19 has impacted the global construction market with supply chain disruptions, workforce restrictions, and legislative changes [7]. Unlike other sectors and industries, the construction industry could not implement telecommuting to mitigate the safety challenges and productivity disruptions associated with the pandemic [8]. IOIM may face a series of risks, such as shortage of human resources, broken capital chain, inability to carry out field tests, and inability to transform research results, which will ultimately lead to the failure of innovation. In early November 2020, the Sichuan-Tibet Railway in China was fully under construction. The Sichuan-Tibet Railway is considered a world-class challenge, requiring IOIM to make technological breakthroughs first. For example, solve the problems of personnel oxygen deprivation and machinery transportation when constructing in ultra-high-altitude areas; ensure the safe construction of extra-long tunnels in high ground stress and earthquake-prone areas. However, affected by the epidemic, the innovation organization faced multiple obstacles. The original research schedule was stretched. Increased uncertainty led to frequent changes in the participants of the innovation organization. The inability to communicate face-to-face reduces the bonding strength of innovation cooperation networks. If innovation is not guaranteed, the project will face inestimable overtime and overcost. This attracted the attention of the project managers, and a special research fund was approved, which this study was supported by. The purpose of this study is to find management methods for innovation organizations so that they can survive and achieve their goals in a crisis.

To achieve the above, the concept of resilience was introduced, and it is held to be a very promising concept to explain how organizations can survive and thrive amidst adversity or turbulence [9, 10]. This brings new management concepts to innovation organizations. The risk management of innovation projects in the past will not be effective in the face of emergencies [11]. Increasing the focus is moving from looking at tools to assist in crisis response to tools that contribute to improved preparedness before a crisis hit [12]. Ruiz-Martin et al. [13] proposed that organizational resilience is considered a property, ability, or capability that can be improved over time. However, they did not find consensus about the elements that contribute to improving the level of organizational resilience and how to assess it. According to Barasa et al. [14], the resilience of organizations was influenced by the following factors: material resources, preparedness and planning, information management, collateral pathways and redundancy, governance processes, leadership practices, organizational culture, human capital, social networks, and collaboration. Lengnick-Hall et al. [15] hope to improve organizational resilience through strategic human resource management. Organizational resilience is a multi-tiered concept [16]; however, most of the current research is based on its underlying factors of it. At present, the practical application value of resilience management is not well reflected; especially, the research on resilience for IOIM is still in its infancy.

To this end, we hope to make contributions in the following areas:

1. Define the concept of organizational resilience of IOIM. This work is necessary because the purpose of this study is to improve organizational resilience, so the basic definition of resilience will provide the direction for efforts. Although the concept is considered promising, it has been criticized for being vague and lacking a consistent definition, thus reducing the significance of the concept for practice and research [17]. Resilience was generally taken to mean a system's ability to continue to meet its objectives in the face of challenges. The concepts of resilience that were used in this paper emphasized not just a system's capacity to withstand shocks, but also to adapt and transform. According to the attributes of organizational resilience, we divide it into two dimensions, which also provide a concise path for the subsequent application of the structural equation model.
2. Examine the impact of cooperative network characteristics on organizational resilience. Combining organizational resilience with social network theory, hypotheses on the relationship between cooperative network characteristics and organizational resilience are proposed, and structural modeling methods are applied to verify these hypotheses. The findings show that there is a positive correlation between network characteristics and organizational resilience. Furthermore, the strength of network connections has a direct impact on the preventive and resistance ability of resilience. Meanwhile, network heterogeneity has an impact on the dual ability of resilience. A strongly connected network with a certain heterogeneity needs to be constructed to improve organizational resilience.
3. Propose resilience enhancement strategies of IOIM. The impact of collaborative network characteristics on organizational resilience has been demonstrated, and then the resilience can be enhanced based on the impact path. Qinghai-Tibet Railway is presented as a representative IM case in this study, and its innovation organization is relatively resilient. By analyzing the characteristics of an innovation organization cooperation network, we can propose a preliminary resilience management strategy, such as strengthening the connections and changing the central position of the participants to improve the resilience of the overall IOIM.

This paper is organized into six parts. The concepts and constructs of organizational resilience and innovation networks are first discussed by employing a systematic literature review. The second part details the research design and gives the hypothesis. The third part describes the research method. The forth reports data collection and analysis. The fifth discusses the findings and suggestions. The final part provides the concluding remarks.

## 2. Literature Review

### 2.1. IOIM

Innovation organizations of infrastructure megaprojects (IOIM) defined from the perspective of management refer to the activity system with human elements formed to achieve the innovation goal of a specific construction project, which keeps close contact with the complex external environment. Because of the characteristics of the construction industry, IOIM focuses not only on products, but also more on the production process [18]. As Chen et al. [19] pointed out that due to socio-technical complexity, project uniqueness, and triple constraints of megaprojects, the traditional innovation management mode is no longer applicable. The key lies in the collaborative innovation of stakeholders in the organization. Ozorhon's research suggests that the innovation process of construction projects requires the joint participation of clients, contractors, subcontractors, suppliers, consultants, and designers [20]. In addition to the research on innovation process of IOIM [21], innovation influencing factors [22, 23] and innovation performance [24] evaluation are also research hotspots in recent years.

**Figure 1:**
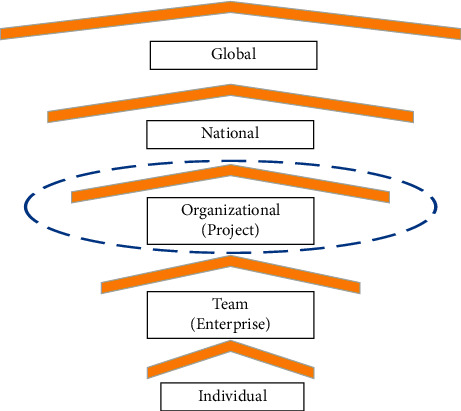
Innovative organization levels.

By discussing innovation activities at the project level, we can get a broader perspective to put forward management countermeasures [2, 25]. According to different research objects, the level of innovation organization can be divided into five levels (Figure 1). As IOIM involves multiple public and private stakeholders, we limited our research to level 3. Innovation based on a complex megaproject needs to be managed from a more macro perspective in a network of interdependent participants [26].

### 2.2. Resilience of Innovation Organization

The innovation organization may be a single institution or any institution among the project stakeholders, yet they all face the possibility of innovation failure, and even well-progressing innovation projects can be halted due to funding issues [27]. Ren and Bao [28] point out that innovation organizations are always at political, economic, social, and natural environmental risks, and many innovation projects encounter suspension or failure during the stages of cooperation and coordination. Unlike other studies explaining the causes of innovation failure, Azim et al. [29] focused on team behavior as the cause of innovation failure. Oeij [30, 31] defines the behavior of innovation resilience and applies theories related to human resource organization management to help innovation organizations achieve resilience and increase the chance of innovation success. Granig and Hilgarter [32] emphasize the use of proactive resilience strategies to improve the sustainable viability of organizations. This study also looks at innovation from the perspective of organizational resilience.

Bothello and Salles-Djelic [33] argue that the key capability underpinning sustainable innovation in an organization is the ability to deal with risk and respond to uncertainty to prevent, resist, and recover from disruption. Reinmoeller and Van Baardwijk [34] studied the innovation strategies of a group of multinational companies, arguing that diversity of innovation strategies can maximize the company's chances of successfully responding to and adapting to crises; that is, it creates resilience. Akgün and Keskin [35] examined the effects of organizational resilience-related variables on firms' product innovation capability and performance through an empirical study of 112 enterprises. Lv et al. [36] conceptualized organizational resilience for innovation, extracted key elements of innovation-related organizational resilience from existing literature, and tested them through multiple case studies.

In general, few studies discuss the combination of innovation management and organizational resilience. Existing research objects are often targeted at specific firms rather than complex innovation organizations composed of multiple firms. Therefore, it is necessary to study the relationship between the characteristics of multi-firm innovation cooperation networks and organizational resilience, to identify the sustainability of innovation and seek the optimization path of resilience.

### 2.3. Innovation Network

Social network theory has been widely used in a variety of disciplines [37], such as sociology [38], information science [39], engineering [40], biology [41], and linguistics [42]. Applying social network theory to innovation organizations can explore the complex behavior among partners in innovation networks [43]. A network of distributed partnerships with a common innovation goal is called an innovation network [44].

Keast and Hampson [45] explore roles in innovation networks and provide management strategies using the Collaborative Research Centre for Engineering Innovation as an example. Landsperger et al. [46] analyzed the impact of network managers on the performance of innovative network relationships. Lazer and Friedman [47] found through computer simulation that ineffective networks are more conducive to exploratory innovation than effectively closed networks. Uzzi and Spiro's research [48] found that small-world networks are conducive to the flow of information within the organization, promote knowledge sharing among individuals, and thus improve organizational innovation performance. Razak and Saad [49] used qualitative and case study methods to examine the role of universities in the cultural evolution of the triple helix of innovation networks.

However, these studies mostly stand from the perspective of ego-network analysis and pay more attention to how individual behaviors are affected by network members. It is impossible to analyze the overall structure of the network only by examining the influence of the structural characteristics of the cooperation network between individuals within the organization. For IOIM, its innovation network has a clear network boundary and definite network constituent subject, which is suitable for analysis from a global-network perspective. Existing empirical studies mostly take innovation performance as an explanatory variable [50, 51] and lack of investigation into the process performance of innovation organizations responding to crises.

The summary in Table 1 indicates there is a research gap in the resilience of IOIM. More specifically, most studies did not consider the relationship between innovation network structure and resilience. In some studies, even though the relationship is considered, it is not fully explained. For example, Omer et al. [53] proposed a methodology for assessing resilience by using social network analysis, but this direct application is lacking in empirical evidence. Each kind of network has its particularities, and its resilience cannot be uniformly measured in one way. Before applying network features, we need to determine if they are applicable.

## 3. Theory and Hypothesis

### 3.1. Organizational Resilience Dimension

The notion of resilience has become increasingly important to all organizations, and organization theory currently does not reflect its importance. Although the theory of organizational resilience is still under development in some aspects, scholars have reached a consensus on the mechanism of organizational resilience. Organizational resilience theory provides insight into how organizations and the individuals and units of which they are comprised continue to achieve desirable outcomes amidst adversity, strain, and significant barriers to adaptation or development [55].

Organizational resilience is recognized by researchers as a capability of an organization, and as a subject, organization uses this capability to survive in a complex environment. Organizational resilience is not only a multi-level concept but also a multi-dimensional concept, which is the result of the interaction between the organization and the external environment. It has the characteristics of a dynamic system and is also a measurable socio-cultural concept and paradigm. When it comes to organizational resilience, due to the vagueness and uncertainty of the definition of the concept, there are great differences in the classification and measurement of organizational resilience dimensions [17].

Williams et al. [56] argue that resilience can be improved by strengthening organizational crisis management and recovery ability. Tasic et al. [57] explore the factors that influence crisis preparedness and response at the individual, organizational, and environmental levels, as well as the learning process of strengthening organizational capacity to enhance resilience. According to the resilience process, Duchek [58] divided organizational resilience into preventive ability before adverse events, coping ability when adverse events occur, and adaptive ability after adverse events occur. Andersson et al. defined four characteristics of resilience, including risk awareness, priority cooperation, agility, and improvisation [59]. According to previous studies on organizational resilience, a resilient organization must have the ability to maintain the stability of the overall system and the ability to optimize and improve the system. Buliga et al. [60] propose that robustness and adaptability represent the two poles on the resilience continuum. Lv et al. [36] proposed the concept of dual innovation resilience from the perspective of stability and adaptability. Here, drawing on his research, resilience is divided into the ability to resist and recovery adaptability.

The capability to prevent and resist is reflected before and during the crisis, which is the state before the overall function of the organization system reaches the lowest point. The capability to recover and adapt is reflected after the occurrence of the crisis, which is gradually improved after the system function reaches the lowest point. The capability of prevention and resistance is closely related to the capability of recovery and adaptation. Organizations that are willing to see potential threats are often able to take the active response and recovery plans [61]. Stability and adaptability are integral parts of resilience, which sheds light on constructing and measuring resilience from two primary dimensions. From the duality view, stability and adaptability are interdependent and mutually reinforcing, and resilient organizations have both capabilities [36]. The disturbed system goes from stable to fluctuating and then to a new stable until the next disturbance occurs and so on. In this study, we paid more attention to the performance of organizational resilience within a cycle; that is, following the rule of time, organizational resilience before disturbance has an impact on the one after disturbance. Therefore, we propose that within the resilience dimension, the preventive and resistant capability of innovation organizations has a positive impact on recovery adaptability:

**Hypothesis 1 .**

Prevention and resistance capability of innovation organizations has a significant positive impact on recovery and adaptation capability.

### 3.2. Network Characteristic Dimension

Social network theory is a useful methodology to model and study the structural and communicative relations in organizations. The idea is to represent an organization as a network composed of the organization's staff (represented as nodes in a graph) and the relations between them (represented as links). The participants of IOIM and their relationships are major elements in the innovation network. Nodes can be individuals, enterprises, technologies, or patents [62], and links can represent the transmission of knowledge, information, or resources [63]. This representation allows us to answer the following: which node or link is the most critical in the network [64]? The answer to this question can be used to measure the resilience of the network, but also the resilience of the organization [65]. The network characteristic measurement indicators often involved in empirical research include centrality, density, cohesion [66], clustering coefficients, degree distribution, and correlation [67]. These indicators express the main network characteristics by quantitative means. However, due to the singleness of megaprojects, the innovation organization networks of megaprojects are also different, which often produces great differences. Therefore, it is impossible to determine the resilience of organizations by establishing unified quantitative indicators. This requires the use of qualitative methods to explain the relationship between network characteristics and organizational resilience and then combined with quantitative methods to analyze. In this paper, network characteristics are divided into network connection strength and network heterogeneity.

#### 3.2.1. Connection Strength and Resilience

Here, network connection strength includes the concepts of degree and density, which describes the degree of interaction between network components, that is, the average degree of interaction between participants in the network. Coleman [62] pointed out that when the degree of interaction between actors is high, the exchange of information and resources between actors will also increase. The management of information can determine whether an organization is resilient [68]. Wehbe et al. [69] pointed out that the organizational network density of engineering projects is positively correlated with risk prevention, risk response, and recovery. Strong linkages among participants facilitate enhanced collaboration. A strong connection can promote the generation of trust relationships [70]. Trust between organizations expresses the belief that their vulnerability will not be taken advantage of by other parties, which will ease the confrontation and defense behavior between each other and enhance the sense of cooperation [71]. Strong relationships also enhance resilience in dynamic dimensions. Close relationships enable organizations to deal with uncertainty in a timely and effective manner [72].

In the process of construction and innovation of megaprojects, it is necessary to keep close contact between the participating units to ensure that they are informed of the work progress of partners and the actual development of the whole project at any time, to enhance the ability to resist risks. The strength of network connection is generally used as the evaluation of system vulnerability. The system with lower vulnerability has stronger resilience and resistance. For example, A and B can share the risk of technological innovation with high connection strength. Therefore, the strength of network connections may affect resilience by affecting the organizational structure. Hence, Hypotheses 2 and 3 are proposed:

**Hypothesis 2 .**

The connection strength of innovation organization network has a significant positive impact on the prevention and resistance capability.

**Hypothesis 3 .**

The connection strength of innovation organization network has a significant positive impact on the recovery and adaptation capability.

#### 3.2.2. Heterogeneity and Resilience

Network heterogeneity can be understood as the diversity of network subjects, which is also the main reason for the uneven distribution of networks and the difference in node centrality. Higher ranking members of the network will seek information from the network in large proportion, compared to lower ranking members who will seek information from fewer companies [66]. If the network aggregation degree is high, it represents poor heterogeneity, then the interaction of the organization is relatively centralized, and the interaction of a few key nodes represents the entire organization [73]. Therefore, the higher the heterogeneity, the stronger the flexibility of the organizational structure. After the main node is damaged, it can still recover quickly and adapt to the new environment.

Flexible networks generated by diverse actors can create resilience in the field of sustainability science, where optimal solutions can be found in the search for stability and adaptability [74]. With the increase in project complexity, it is difficult to accurately predict and analyze risks only by individual subjects, and diversified partnerships have a positive correlation with risk prevention and resistance [75]. For complex projects, we rely more on networks of disparate but interconnected actors, which makes it possible to deal with high levels of uncertainty in dynamic environments [76].

Carayannis et al. [77] proposed that organizational design is a way for organizations to achieve sustainable development, and heterogeneity can be strategically utilized for design practice. In innovation research, individual-level heterogeneity mainly refers to the heterogeneity of job or task-related attributes, such as occupation, education, knowledge, and skills [78]. According to the level of innovation organization targeted by this study, network heterogeneity is mainly reflected in the different attributes of cooperative enterprises in the innovation network. For example, the owner and contractors can jointly participate in the same innovation project but undertake different tasks in the project implementation. In summary, the following hypotheses are proposed:

**Hypothesis 4 .**

The heterogeneity of innovation organization network has a significant positive impact on the prevention and resistance capability.

**Hypothesis 5 .**

The heterogeneity of innovation organization network has a significant positive impact on the recovery and adaptation capability.

### 3.3. Conceptual Model

**Figure 2:**
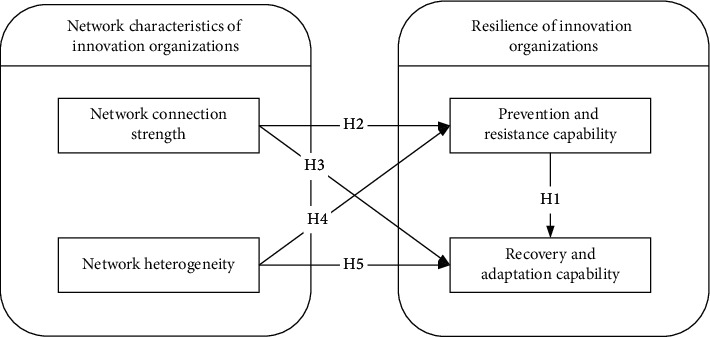
Research framework and research hypothesis.

Based on the hypothesis proposed above, the conceptual model is shown in Figure 2. This conceptual model demonstrates the impact of cooperative network characteristics (including network connectivity strength and network heterogeneity) on the organizational resilience of innovation organizations.

## 4. Methods

Social network analysis can be divided into two basic perspectives: relationalism and structuralism. The theoretical basis mainly comes from the ideas about relational embedding and structural embedding proposed by scholars such as Granoveter [79, 80] and Burt [81]. From the perspective of relationalism, scholars mainly focus on how the social connection between actors affects the specific behavior and process.Studies of the social networks include the content, type, intensity, and persistence of the relationship. The structuralist perspective focuses on the location orientation of actors, emphasizes the understanding of actors' behavior from the social structure reflected by the modular relationship between two or more actors, and discusses the relevant role of the network. This kind of research regards networks as a topological structure. Although some progress has been made in innovation studies based on structuralism [66, 82, 83], the resilience behavior and results of innovation organizations for megaprojects may be quite different even if the innovation subjects are in the same position in the network. Therefore, analysis from the perspective of relationalism is a useful complement to structuralism.

**Figure 3:**
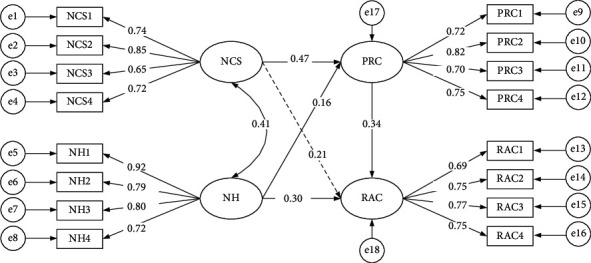
Standardized output of the hypothetical model.

According to the conceptual model in Figure 3, what we want to study is the relationship between structural attributes and capability attributes in innovation organizations. As mentioned above, it is currently impossible to judge resilience through network analysis alone, which requires continuous data accumulation. We must first prove the correlation between structure attributes and ability attributes, which is still a research gap. Therefore, we adopt the method commonly used in social science to conduct research from the perspective of relationalism. In subsequent studies, we will apply social network analysis to explore strategies to enhance resilience.

As a multivariate statistical technique, the structural equation model (SEM) can deal with the relationship between multiple causes and multiple results at the same time, allow the measurement error of variables, and measure the degree of fitting of the whole model. In consideration of the interaction between variables in this study, the SEM method was used for research.

### 4.1. Survey and Data Collection

The introduction introduces the research background. Restricted by sample acquisition and funds, the survey objects mainly focus on China's complex railway projects. All of the surveyed institutions have participated in infrastructure megaprojects such as Qinghai-Tibet Railway, Beijing-Tianjin Intercity Railway, and Beijing-Shanghai High-Speed Railway. China continues to overcome world-class technical problems in the construction process of IM and brings huge spillover effects of technological innovation through the promotion and application of similar projects. Therefore, this research is representative and advanced. Technology innovation of IM is a multi-factor, multi-subject, multi-stage integration and coordination process. To ensure the coverage of the questionnaire and make the survey representative, the respondents of the questionnaire are all subjects from the innovation organization network, including government agencies, owners, universities, research institutes, designers, contractors, and suppliers. Their number and proportion are shown in Table 2. Taking Qinghai-Tibet Railway as an example, IOIM here mainly includes government departments such as the Ministry of Railways, universities such as Beijing Jiaotong University, research institutions such as the Chinese Academy of Sciences, and contractors such as China Railway Construction Corporation Limited.

The questionnaires were distributed and collected from October 2021 to December 2021, and the respondents responded to the questionnaires through e-mail and WeChat. In this study, a total of 190 questionnaires were sent out, and 164 valid questionnaires were received, with an effective recovery rate of 86.32%. The number of valid questionnaires could meet the requirements of model testing.

### 4.2. Variable Measurement

Limited by the size of the research object, it is difficult to describe relevant variables through intuitive observation or public data, and subjective measurement indicators need to be set to measure them. Since all potential variables have measurement errors, each potential variable must be estimated by more than two measurement indexes, so this study will set at least three measurement indexes for each variable requiring measurement [84]. Based on the literature review, combined with the characteristics of IOIM, a 5-point Likert scale with anchors ranging from (1) “strongly disagree” to (2) “strongly agree” was used to form the measurement items of the variables involved in this study (Table 3). Since most of these measurement indicators come from existing literature and have been empirically tested, the reliability and validity of these measurement indicators can be guaranteed. After the scale was formed, we took the opportunity to project cooperation with large railway construction enterprises and regulatory authorities, consulted with personnel with many years of work experience, determined the applicability of the measurement items, and formed a survey questionnaire.

## 5. Results

### 5.1. Descriptive Statistics

Table 4 presents the means, standard deviations (SD), and correlations. It shows that most of the correlations were positive and significant at the level. The assessment of normality test (skewness and kurtosis) provides values for all constructs between <1, demonstrating they meet the acceptable range for normality (e.g., |skewness| < 1, |kurtosis| < 1).

### 5.2. Reliability and Validity Testing

SPSS 26.0 was used to test the reliability and consistency of the questionnaire. Cronbach's alpha coefficient was greater than 0.6 as the criterion, and the reliability of the model was tested. The results are shown in Table 5. The Cronbach's *α* values of each scale all met the requirements, indicating that the scale had high reliability.

Confirmatory factor analysis was conducted on the convergent validity of the proposed model. Further evaluation of the structural model can only be performed if the fitness of the measurement model reaches an acceptable standard. According to statistics, the load value of the standardized factor is between 0.5 and 0.95, indicating that the model fit is good and the scale consistency is high. It can be seen from Table 4 that the standardized factor loads of all variables are above 0.5. Composite reliability (CR) is higher than 0.8, and average variance extract (AVE) is greater than 0.5. Therefore, the questionnaire has good discriminant validity.

### 5.3. Model Fitting and Hypothesis Testing

After testing the reliability and validity of the measurement model, it is necessary to perform a goodness-of-fit test. According to the test results (Table 6), the fit index results show CMIN/DF = 1.266, less than the critical value 3; RMR = 0.075, not meeting the requirement of less than the critical value 0.05; RMSEA = 0.040, less than the critical value 0.08; GFI = 0.916, TLI = 0.973, CFI = 0.978, IFI = 0.979, NFI = 0.906, the above adaptation indices are all greater than the critical value of 0.9; and AGFI = 0.883, which is greater than the lowest critical value of 0.8. Except for a certain deviation between the RMR value and the requirements, the rest of the fit indices are all within a reasonable range. Therefore, it can be considered that the fit of the complex and large-scale infrastructure innovation organizational resilience model constructed in this study meets the requirements of the empirical data, and the fit between the theoretical model and the data is relatively high.

The structural relations among latent variables, estimated values of standardized path coefficients, *T*-values, and hypothesis testing results are shown in Table 7. Among the 5 hypotheses proposed above, Hypotheses 1, 2, 4, and 5 have passed the verification and are supported.  Hypotheses 2 and 5 are significant at the level of confidence *α* = 0.01. However, the Hypothesis 3 path coefficient is not significant at the level of confidence *α* = 0.05, so the hypothesis is not supported. The actual model and path coefficients are shown in Figure 3.

### 5.4. Analysis of Empirical Results

#### 5.4.1. Innovation Network Resilience Follows the Law of the Adaptive Cycle

From the test results, Hypothesis 1 is established, indicating that there is an influence between the resilience attributes in the innovation network, and follows the adaptive cycle (growth/exploitation-conservation-release/collapse-reorganization). That is, prevention and resistance capability has a positive impact on recovery and adaptation capability. Some ecologists believe that this relationship should be negatively correlated, but these arguments lack systematic limitations. The object of this study is IOIM, and the innovation partners constitute a social system conforming to the characteristics of a small-world network. The component units of this system are relatively stable, and the source of resilience attribute mainly exists in the connection between individuals and their position. Through the empirical analysis, we prove that preventing and enhancing the capability to resist the crisis in the early stage have a positive impact on the recovery in the later stage. This is because the innovation process is a complex adaptive system, and the process of competition and cooperation among various innovation elements (such as technology, strategy, market, culture, organization, and system) is also a process of coevolution.

#### 5.4.2. The Connection Strength of Innovation Network Is the Main Influencing Factor of Prevention and Resistance Capability

In terms of network connection strength, Hypothesis 2 is supported and Hypothesis 3 is not supported; that is, network connection strength has a significant positive impact on prevention and resistance capability, but has no direct impact on recovery and adaptation capability, and there is an indirect impact path. The possible reason is that the prerequisite for the existence of connection strength is that there is a connection between nodes. Knowledge, human resources, capital, and other innovative resources are the blood to maintain the operation of the innovation network. The faster the innovation resources are generated, flowing and circulating in the network, the stronger the ability to resist risks. However, after a crisis, the connection between nodes may be cut off, and the premise of recovery is to provide the supply path of resources, while the connection strength is not the primary consideration. Therefore, according to the above conclusions, it is necessary to input and recycle good innovation resources to build an innovation network with resilience. Enterprises, universities, and other innovation partners need extensive communication and coordination. Policies, institutions, and benefit distribution mechanisms provide necessary conditions for the increase of network connection strength.

#### 5.4.3. The Heterogeneity of Innovation Network Is the Main Influencing Factor of Recovery and Adaptation Capability

The results show that Hypotheses 4 and 5 are supported, and the normalized path coefficients are 0.16 and 0.30. The results indicate that network heterogeneity has a positive impact on both resilience capabilities and has a more significant impact on the recovery and adaptation capability. This result proves that the multi-layer and complexity of innovation networks should be fully considered in the construction of resilience. Different from traditional network theory, which believes that network connection is random, the connection of the innovation network is a conscious process and has a certain preference. Nodes tend to choose to connect with nodes with strong capabilities, great influence, good social reputation, and advanced technology, thus becoming key roles in the network. The heterogeneity of the network, such as centrality, can explain whether the participant plays the role of a “bridge” in the innovation system. The participant with high centrality is the intermediary of information and knowledge dissemination, and it has the opportunity to access more heterogeneous information. Before a crisis, heterogeneity can concentrate superior resources to cope with emergencies and maintain the stability of the system. After the crisis, to meet the timeliness requirements of the recovery, the key role as the core part of the innovation network, whether it can effectively organize and connect all kinds of nodes in the network becomes the key to the recovery action. After returning to the previous state, the input and flow of superior resources also become the driving force of innovation performance improvement. To sum up, the formation of a “differential pattern” in innovation network is the basis for improving resilience capacity, and it is necessary to configure and guide the collaborative activities of innovation partners through policy formulation.

## 6. Discussion

Innovative tasks are among the riskiest endeavors in organizations [95]. This is especially true for infrastructure megaprojects, where a key technological innovation often determines the success or failure of the project. Therefore, the organizational resilience of IOIM must be improved. However, there are few types of research in this field. The conclusion of this study can enrich the relevant theories in this research field and provide practical guidance.

### 6.1. Theoretical Implications

There are three key theoretical and empirical implications of the above results. First, organizational resilience theory can be used to guide IOIM to deal with uncertainty in the innovation process. The theory of organizational resilience emphasizes that resilience is a multi-dimensional set of capabilities. Through questionnaires, we measured different dimensions of organizational resilience and obtained the correlation between different resilience capabilities. This study suggests that improving prevention and resistance capability before a crisis can help the development of recovery and adaptation capability. Second, taking social network theory as the theoretical basis, this paper's hypothetical model was verified and supported by empirical data. It is proven that the characteristics of innovation networks have a direct impact on resilience. For IM, strategic organizational structures need to be prioritized, with innovation organizations focusing more on the flow of knowledge and resources. This provides us with a broad framework for managing resilience; that is, by exploring and changing the characteristics of innovation networks we can obtain more resilient innovation organizations. Finally, this pioneering empirical study tested a relationship model between structural attributes and capability attributes. Unlike engineering entities, innovation organizations cannot measure resilience through specific network simulations. It is difficult to provide an IOIM management plan based on factors only identified at the individual level. Thus, this research contributes empirically to integrating innovation studies at the macro level with resilience capabilities studies at the micro-level and enriching the academic literature on organizational resilience.

### 6.2. Managerial Implications

Through the research results, we clarified the impact path of resilience and verified the influence of network connection strength and heterogeneity on the resilience of innovation organizations by using the SEM method. Further, we can select the relevant network characteristic parameters of innovation organizations, analyze the cooperation characteristics of successful engineering innovation cases, and then guide the innovation management of other projects.

Most management studies require empirical studies [96], and organizations need different resilience strategies in different risk stages. Here, suggestions for practice are given in a clear form as shown in Table 8.

**Figure 4:**
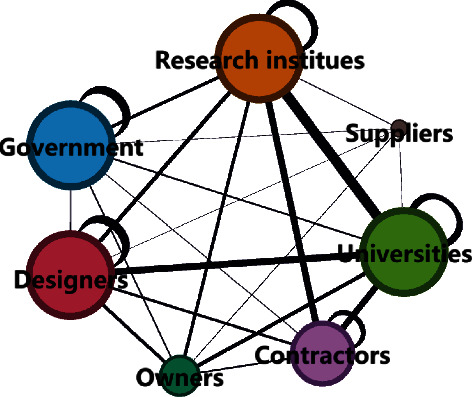
Innovation network of Qinghai-Tibet railway reconstructed by the group.

Here, we take the Qinghai-Tibet Railway as an example to illustrate. The Qinghai-Tibet Railway is a representative of infrastructure megaprojects, which have generated many intellectual property rights and scientific technological achievements, the most prominent of which is to solve the three problems of permafrost, alpine hypoxia, and environmental protection. As a reference case, we first draw the cooperation network of innovation organizations through the cooperation frequency of innovation participants. Secondly, quantitative indexes describing network characteristics are selected. The average path length and node degree can be selected for network connection strength. Heterogeneity can select degree centrality, intermediate centrality, and other parameters. After classifying innovation partners by category, corresponding parameter results of network characteristics are obtained from calculation (Figure 4): (1) the connection strength of research institutes, designers, government departments in charge of industry, and universities is relatively high; (2) the centrality of owners is lower than that of the construction contractor, so it can be seen that the influence of adjacent nodes is lower; (3) in the innovation cooperation network, designers and government authorities are connected with important enterprises, and they generate large network heterogeneity.

**Figure 5:**
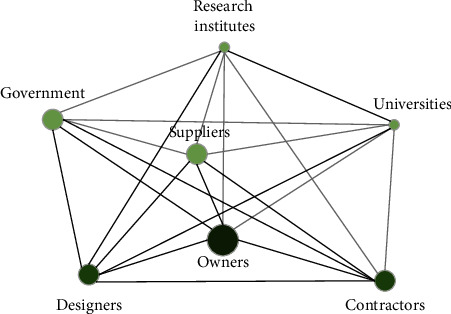
Random innovation network.

**Figure 6:**
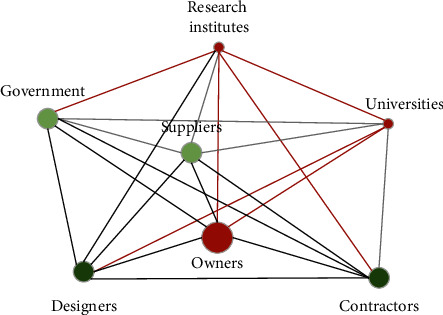
Reconstructing random innovation network.

According to the conclusions above, network connection strength and network heterogeneity have a significant positive impact on prevention and resistance capability, and network heterogeneity has a significant positive impact on recovery and adaptation capability. Combined with the feature of the Qinghai-Tibet Railway network, we can reconstruct the random innovation network (Figure 5) in the following aspects: (1) in terms of network scale, the number of participating units should be controlled within a reasonable range. Construction contractors and universities should not be too few, and they are easy to form a trusting relationship; designs and government departments should not be too many; otherwise, it is easy to cause command, and information and instruction transmission errors will hinder communication. This aspect is reflected in the node degree of owners, government and designers will increase obviously, but the intermediary effect between contractors and universities is not obvious; (2) the relationship between research institutes and contractors, research institutes and universities, and research institutes and government should be strengthened. It is also necessary to strengthen the relationship between designers and universities, owners and research institutes, and owners and universities. These reinforcements are shown in the red border in Figure 6; (3) the degree of centrality of the designer should be ensured. On this basis, strengthen the degree of centrality of research institutes and universities, and the intermediary centrality of owners. The above aspects are represented in the red nodes in Figure 6. Comparing the reconstructed network with the innovation network of the Qinghai-Tibet Railway, the network structure and node relationship are the same. According to the proven relationship between innovation network characteristics and organizational resilience, the resilience of the random network is improved.

To sum up, here are some suggestions for other infrastructure megaprojects: (1) to enhance the resist capability, the transmission cycle of knowledge flow in the overall scientific and technological innovation cooperation network should be strengthened. Focus on strengthening communication between scientific research institutes, designers, government authorities, and universities, and achieve innovation goals based on information sharing and resource sharing; (2) to enhance the resilience of adaptability, it is necessary to strengthen the central role of government industry departments and owners, and give play to the ability to concentrate on major tasks. The core organization in the network should play a coordinating role and establish a reasonable incentive and withdrawal mechanism; (3) to enhance the overall resilience, it is necessary to strengthen the depth of participation of designers, universities, research institutes, and contractors. A partnership contract should be signed to restrain all parties involved and share risks and benefits.

## 7. Conclusions

Earlier studies were more concerned with innovation performance and neglected aspects of risk management. Of course, improving performance level is an important goal of innovation organizations, but the prerequisite of good performance is to avoid failure. The hypothesis of the organizational resilience dimension in this study is supported, which not only verifies the relevant theories of organizational resilience but also changes the focus of innovation organizations from performance to resilience. Among the few studies on IOIM, no paper has explored the role of organization resilience, and this study can fill in the gap.

In addition, from the perspective of systems thinking, this study reveals the relationship between network characteristics and organizational resilience of innovation. Specifically, the IOIM is in a complex internal and external environment, which requires sustainable innovation through organizational management design. However, the management system is still a black box at present. How to motivate innovation partners? How to achieve innovation goals after the crisis? There is not enough theoretical support for these issues to be studied. It can be seen from the above research conclusions that network characteristics are significantly correlated with organizational resilience of innovation, and the pointing out of this relationship provides support for applying social network theory to management decisions at the system level. By studying the mechanism of the characteristics and resilience of innovation organizational network, the organization can obtain adaptability in crisis and finally achieve the innovation goals of infrastructure megaprojects under uncertain conditions.

Besides the theoretical importance of the findings, this study has high practical relevance as the termination of innovation projects is regularly occurring in organizational innovation activities [97]. Therefore, attention should be paid to building a network of partners for IOIM, so that innovators can be prepared for possible setbacks (such as the termination of innovation projects) and can quickly recover from shocks and adapt to the new environment.

It should be noted that the questionnaire used in this study is based on theoretical research and there is no ready-made scale, so relevant questions should be as simple and clear as possible. To avoid the inaccurate wording of the questionnaire or the distortion of the survey data caused by excessive academics, the questionnaire has been modified several times. In the questionnaire, respondents were reminded to answer according to their experience in participating in innovation projects, and there is no right or wrong result. The empirical test results were obtained by factor analysis, reliability test, validity test, and other steps, which met the requirements of SEM method. Due to the availability of samples, the railway industry is selected for this study, but the survey is also applicable to infrastructure fields outside the railway industry.

The limitations of this paper are mainly reflected in the following aspects. First, based on organizational resilience and social network theory, this paper analyzes the impact of the strength and heterogeneity of innovation networks on resilience. However, there are other factors that affect organizational resilience of infrastructure megaprojects, such as external environment, resource reserve, and organizational size. Therefore, the conceptual model of this study does not cover all the influencing factors.

In addition, the questionnaire of this study is distributed through the Internet, which is a nonprobability sampling and prone to sampling bias. In other words, most respondents are Internet users and cannot represent the participants of all in IOIM, which makes the conclusions of this study have certain limitations.

Finally, more theoretical models and influencing factors should be selected in subsequent studies to build a more comprehensive theoretical framework. It is necessary to carry out strict random sampling survey to avoid sampling deviation. In addition, more practical data need to be collected to verify the more complex nonlinear relationship between network characteristics and resilience. Based on the existing research results, future research can explore the impact of social network analysis indicators on innovation resilience. Collect more project data and use a combination of qualitative and quantitative research methods to further reveal the impact of different types of participants and different management modes on resilience.

## Figures and Tables

**Table 1:** Related study comparison.

Authors	Organization	Methods	Objectives	Whether considering structural characteristics	Whether considering resilience capability
Ozorhon [20]	Project-based innovation	Case study	Investigate how innovation occurs in construction project settings	No	Yes, but not explicit
Wang et al. [52]	Innovation network	Social network analysis	Explore how absorptive capacity acts on innovation performance	Yes	Yes, but just absorptive capacity
Omer et al. [53]	Organizational system	Social network analysis	Propose metrics for measuring resilience	Yes, but lack of empirical evidence	Yes, but the classification is unclear
Yang and Hua [54]	Innovation network	Literature review	Put forward the theoretical framework of sustainable innovation organization	Yes	No
Bowers and Khorakian [11]	Business company	Case study	Propose a theoretical framework that combines the generic innovation process with project risk management	No	Yes
Calik et al. [6]	Innovation network	Literature review	Propose a conceptual model that shows all key factors of sustainable innovation	No	Yes, but not explicit
Ning and Gao [25]	Project-based innovation	Case study	Examine how the resilience framework deals with explorative quality management (EQM) in innovative building projects	No	Yes
Lo and Kam [18]	Architecture, engineering, and construction (AEC) industry	Literature review and conversation interview	Innovation performance evaluation for the AEC industry	No	No

**Table 2:** Descriptive statistics of data.

Item	Number	Percentage
Government agencies	27	16.5
Owners	20	12.2
Designers	36	22
Contractors	15	9.1
Research institutes	26	15.9
Universities	20	12.2
Consultancy	11	6.7
Suppliers	9	5.5
Others	0	0

**Table 3:** Measurement of variables.

Variable	Item	Reference
Network connection strength (NCS)	(1) The IOIM that your organization participates in has a large number of stakeholders	Chen [85]; Pryke et al. [86]; Chen and Li [87]
	(2) During the implementation of innovation projects, the communication between various participating units is deep	
	(3) During the implementation of innovation projects, there are frequent exchanges between research units	
	(4) In the innovation cooperation network, various tools such as e-mail, conference, and telephone are used to communicate with each other	

Network heterogeneity (NH)	(1) The IOIM that your organization participates in involves multidisciplinary experts	Wasserman and Faust [88], Sutcliffe and Vogus [89], Carvalho et al. [90]
	(2) There are many different types of partners in IOIM	
	(3) Your organization pays attention to the maintenance of relations with specific categories of research units	
	(4) Other participating units may receive outside assistance through an organization in responding to adverse events	

Prevention and resistance capability (PRC)	(1) Your organization can effectively evaluate the degree of potential risk in a project	Chowdhury and Quaddus [91]; Hillmann et al. [92]
	(2) Your organization develops contingency plans for organizational crises, such as technological innovation fail	
	(3) Your organization is well prepared for unexpected events such as outbreaks, terrorist attacks, and cyber-attacks	
	(4) When emergencies or risks occur, your organization can respond comprehensively and effectively	

Recovery and adaptation capability (RAC)	(1) When a crisis occurs (e.g., when a technology is too slow to be put into practice), your organization can accurately assess the damage	Duchek [58]; Karman [93]; Cheese [94]
	(2) Your technical innovation organization needs a relatively short period of time (less than a month) to acquire the resources (such as capital and talent) to recover to the precrisis state	
	(3) Your technical innovation organization can learn from experience promptly to prevent similar situations in the future	
	(4) After a crisis, leaders consult employees about inappropriate decisions or actions in the organization	

**Table 4:** Mean, standard deviation, and correlations.

Variables	Age	Experience	Education	Tenure	Organization type	NCS	NH	PRC	RAC
Age	1								
Experience	0.106	1							
Education	−0.048	−0.013	1						
Tenure	0.051	0.062	0.084	1					
Organization type	−0.048	−0.173^∗^	0.015	−0.040	1				
NCS	−0.099	0.183^∗^	0.031	0.018	−0.102	1			
NH	0.024	0.077	−0.131	0.042	0.077	0.369^∗∗^	1		
PRC	−0.104	0.072	−0.018	−0.005	−0.041	0.474^∗∗^	0.341^∗∗^	1	
RAC	−0.073	0.079	−0.075	−0.043	−0.010	0.401^∗∗^	0.443^∗∗^	0.460^∗∗^	1
Mean	3.000	2.073	2.427	2.579	3.866	2.384	3.592	2.435	1.997
SD	0.985	0.826	0.656	0.607	2.080	1.010	1.047	1.008	0.940

Notes: ^∗^ indicates a significant correlation at the 0. 05 level. ^∗∗^ indicates a significant correlation at the 0.001 level.

**Table 5:** Reliability and validity analysis of measurement model.

Latent variables	Observed variables	Standardized factor loading	Cronbach's *α*	CR	AVE
NCS	NCS1	0.781	0.822	0.8491	0.5858
	NCS2	0.821			
	NCS3	0.668			
	NCS4	0.783			

NH	NH1	0.878	0.879	0.8907	0.6713
	NH2	0.760			
	NH3	0.819			
	NH4	0.816			

PRC	PRC1	0.793	0.832	0.8516	0.5896
	PRC2	0.793			
	PRC3	0.717			
	PRC4	0.766			

RAC	RAC1	0.723	0.823	0.8467	0.5809
	RAC2	0.713			
	RAC3	0.829			
	RAC4	0.778			

**Table 6:** Measuring the fit of the model.

Fitting metrics	Result requirements	Model fit
*χ* ^2^	The less the better	124.052
*χ* ^2^/d*f*	*χ* ^2^/d*f* < 3	1.266
GFI	GFI > 0.9, minimum > 0.8	0.916
AGFI	AGFI > 0.9, minimum > 0.8	0.883
RMR	RMR < 0.05	0.075
RMSEA	RMSEA < 0.08	0.040
NFI	NFI > 0.9	0.906
IFI	IFI > 0.9	0.979
TLI	TLI > 0.9	0.973
CFI	CFI > 0.9	0.978
AIC	Less than saturated and independent models	200.052
CAIC	Less than saturated and independent models	355.847
ECVI	Less than saturated and independent models	1.227

**Table 7:** Hypothesis test results.

Hypotheses	Paths	Estimate	S.E.	C.R.	Sig.	Results
Hypothesis 1	PRC ⟶ RAC	0.258	0.083	3.095	0.002	Support
Hypothesis 2	NCS ⟶ PRC	0.525	0.116	4.529	^∗∗∗^	Support
Hypothesis 3	NCS ⟶ RAC	0.140	0.089	1.572	0.116	Not support
Hypothesis 4	NH ⟶ PRC	0.164	0.070	2.334	0.020^∗∗^	Support
Hypothesis 5	NH ⟶ RAC	0.184	0.055	3.369	^∗∗∗^	Support

**Table 8:** Managerial implications of core issues.

Core issues	Managerial implications	
(1) What is the focus of IOIM resilience management?	The prevention and resistance capability of IOIM before the crisis	
	The recovery and adaptation capability of IOIM after the crisis	

(2) How to build a suitable cooperation network to make IOIM resilient?	Network connection strength strategy:	Network heterogeneity strategy:
	Set common goals for innovation	Managers provide constant guidance and feedback
	Allocate more communication time	Identifying the unique development needs of innovation participants
	Changing the way information is communicated	Increasing the variety of participants
	Create a cohesive atmosphere	Take advantage of the diversity of ideas, experience, and skills in the organization
	Expanding external communication channels	Strengthen the centrality of the convener
	Using institutional means such as contracts and cooperation agreements	Classification of participants to determine their respective task types
	Forming a risk-sharing mechanism	Form incentive and exit mechanism
	Forming a benefit-sharing mechanism	…
	…	

(3) What are the effects of IOIM network elements on resilience dimensions?	According to the standardized path coefficient, network connection strength mainly affects the prevention and resistance capacity dimension of resilience, and network heterogeneity mainly affects recovery and adaptation capability dimension of resilience	
	With the accumulation of data from infrastructure megaprojects, the resilience of an organization can be evaluated through quantified network element indicators in the future	
	Factor index of network connection strength: number of nodes, connection frequency of nodes, strength of connection chain	Factors of network heterogeneity: degree centrality, betweenness centrality, eigenvector centrality, closeness centrality, clustering coefficient

## Data Availability

The survey data used to support the findings of this study are available from the corresponding author upon request.
